# Evaluation of Hand Function Using Relative Motion Extension Concept (with or Without Night Wrist Orthosis) or Dynamic Extension Orthosis for Extensor Tendon Injuries in Zones 4–6—A Randomized Controlled Trial

**DOI:** 10.3390/life15020249

**Published:** 2025-02-06

**Authors:** Vida Bojnec, Jerneja Vidmar, Zvezdana Sužnik, Aleksandra Orož Koprivnik, Milena Špes Škrlec, Maša Frangež, Neža Majdič, Gaj Vidmar, Breda Jesenšek Papež

**Affiliations:** 1 Institute for Physical and Rehabilitation Medicine, University Medical Centre Maribor, 2000 Maribor, Slovenia; 2 Faculty of Medicine, University of Ljubljana, 1000 Ljubljana, Slovenia; 3 Department of Plastic and Reconstructive Surgery and Burns, University Medical Centre Maribor, 2000 Maribor, Slovenia; 4 Valdoltra Orthopaedic Hospital, 6280 Ankaran, Slovenia; 5 University Rehabilitation Institute, 1000 Ljubljana, Slovenia; 6 Faculty of Medicine, University of Maribor, 2000 Maribor, Slovenia

**Keywords:** hand rehabilitation, extensor tendon injury, RME approach

## Abstract

This study aimed to compare outcomes of early active motion (EAM) using the relative motion extension (RME) approach to outcomes of early passive motion (EPM) with a dynamic extension orthosis (DEO) and to evaluate whether the RME-only approach is equivalent to the RME-plus approach. Fifty adults were randomized into one of the three intervention groups receiving the DEO, RME only, or RME plus orthosis. The score of the Jebsen–Taylor hand function test (JTHFT) without writing and QuickDASH at T1, all measures of mobility at T1 and T2, and grip strength were better in the RME-only and RME-plus group compared to the DEO group, whereas the values of Patient Evaluation Measure (PEM) at T1 and T2, as well as QuickDASH score at T2, orthosis adherence, and the patient’s comfort while wearing the orthoses did not statistically significantly differ among the three groups. The RME concept after extensor tendon injuries in zones 4–6 is superior to the DEO protocol in terms of earlier regain of hand function. The DEO and RME protocols were equivalent regarding patients’ adherence and satisfaction with the orthosis. We found no differences in the RME-plus and RME-only protocols, indicating the safe use of the RME-only protocol in single extensor tendon injuries in zones 4–6.

## 1. Introduction

The wrist and hand tendons are the second most commonly injured structures in the hand [1], especially in the working population [2]. They account for 33.2 injuries per 100.000 per year, occurring predominantly in males, with the highest incidence at the age 20–29 years [1,2]. It is a frequent injury in the working-age population that demands 6.5–10 weeks before returning to work at full capacity [3,4,5]. Extensor tendons are injured more frequently than flexor tendons [1,2] due to their more exposed anatomic location [6]. There is a common erroneous belief that they are simple to treat in comparison to flexor tendon injuries [6]. However, the preservation of the tendon’s length is essential for normal tendon balance since the excursion of extensors over the finger is smaller than with flexors, and tendon imbalances can easily result in worse clinical outcomes [7]. Tendon repair must be followed by early rehabilitation with an orthosis, allowing the necessary tendon glide to prevent adhesions and, at the same time, granting enough protection to the reconstructed tendon [8].

Kleinert and Verdan outlined eight zones in the course of extensor tendons, with zones 4, 5, and 6 located over the proximal phalanx, over the metacarpophalangeal (MCP) joint, and the dorsum of the hand, respectively [9]. The choice of postoperative rehabilitation protocol and splinting after the primary surgical repair of the finger extensor tendon laceration depends on the injured zone. Recently published systematic reviews outline three different motion options for zones 5 and 6 as follows: immobilization, early passive motion (EPM), and early active motion (EAM) [10,11,12,13,14,15,16]. The oldest immobilization protocol entails 4–6 weeks of static splinting of the wrist and fingers in the extended position following the tendon’s surgical reconstruction [6]. The disadvantage of static immobilization is the occurrence of adhesions in the healing process [17], with reported unwanted sequelae such as lag of active extension at the MCP joint and loss of finger flexion [18]. The controlled mobilization of the tendon within the permitted arc of motion promotes its intrinsic healing capacity, whereas immobilization blinds the cells to adaptive tissue requirements [19] and is a major culprit in the formation of tendon adhesions [20]. It has been established, first in animal studies and later in humans, that early active tendon mobilization facilitates more substantial tendon repair after injury [21,22]. In the 1980s, two different motion protocols evolved, one acting on the principle of EPM with the dynamic extension orthosis (DEO) [23] and the other following the theory of EAM with different types of orthoses. The mainstream of treatment in hand extensor injury rehabilitation currently is the relative motion extension (RME) approach [24]. The EPM protocol allows for passive gliding of the repaired tendon with the dynamic component of the orthosis on the dorsal side of the hand, facilitating full passive finger extension at the MCP and interphalangeal (IP) joints with the recoil of the elastic bands. Active finger flexion in MCP joints across 30–40° is permitted by a static component of the orthosis on the volar side of the hand, holding the wrist in 30–40° of extension [23]. The relative motion concept was introduced by Merritt [24] and established on the quadriga effect, according to Verdan [25]. The rationale behind the RME concept is the common extensor digitorum communis muscle (EDC) with multiple tendons, giving each finger separate tendons. Splinting the injured finger with 15–20° more extension at the MCP joint in relation to other MCP joints sufficiently releases tension on the surgically reconstructed extensor tendon to enable early controlled active movement of the finger with the RME orthosis in place. The injured finger can be actively moved in all directions within the limits set by the RME orthosis [24]. Initially, the RME orthosis was worn together with the wrist orthosis with 20–25° extension [26]. Further studies confirmed that a wrist orthosis is not essential for tendon protection, and only the RME orthosis was worn during the day, replaced by the wrist–hand–finger orthosis (WHFO) during the night [3,27]. The rationale further evolved in the direction of also omitting the wrist orthosis during the night. This was supported by the evidence gained in a biomechanical cadaveric study by Kanouzi et al., who demonstrated that the use of the RME-only orthosis allows for safe active movement after surgical repair of an injured extensor tendon of the finger, irrespective of the position of the wrist, i.e., without temporary additional wrist immobilization [28]. Hirth et al. reported in their international electronic web-based survey that 47% of respondents used the RME-plus approach (with an additional wrist orthosis) and 44% used both (RME plus and RME only) in their clinical practice. Only 9% used the RME-only approach (without a wrist orthosis), of which only one-half did not add an overnight wrist orthosis to the RME orthosis [29]. A study published by Hirth et al. randomly categorized patients with finger extensor tendon repairs in zones 5 and 6 into two parallel groups, one wearing an RME orthosis during the day and a WHFO during the night (RME day group), and the other wearing an RME orthosis continuously (RME 24 h group). The outcomes such as total active motion (TAM), grip strength, QuickDASH score, and Patient Rated Wrist/Hand Evaluation (PRWHE) scores were similar between both intervention groups. The authors concluded that the decision to include the additional WHFO should be individual according to the patient’s characteristics [30].

Another recent systematic review focused on rehabilitation protocols after zone 4 extensor tendon repair since this zone’s most efficient rehabilitation protocol has not been determined. The study reported outcomes after using the RME approach, the Norwich program [4], and two other unspecified programs. The review concluded that current evidence is insufficient to determine the optimal early active mobilization program, and further studies are needed to assess outcomes for zone 4 extensor tendon repair [31].

Since there is evidence about surgeon and clinic preferences being the primary barriers to implementing the RME approach [32], we, the authors, believe that further well-designed studies are needed to overcome the stated barriers. According to an electronic web-based survey on the international practice of extensor tendon repair management in zones 5 and 6, there were also answers given by three Slovenian occupational therapists not coming from our center who reported using the DEO in the management of extensor tendon repair in zones 5 and 6, while none reported the use of an RME orthosis [32]. Therefore, we, the authors, believe that more real-time clinical evidence is needed to implement the RME approach in local clinical practice. The rationale for comparing the DEO, RME only, and RME plus orthoses was based on prior studies and expert opinion. EPM with the DEO was chosen as the treatment reported by the Slovenian occupational therapists in the study by Hirth et al. [32]. The EAM with RME approach was chosen as the mainstream of current rehabilitation of extensor tendon injuries [24] and due to the paucity of high-level evidence on the RME-only approach [30].

To the authors’ knowledge, no direct controlled evaluation comparing DEO, RME plus, and RME-only approaches for early controlled motion has been described; this study will provide novel evidence on the rehabilitation of extensor tendon injuries in zones 4–6. There is also a scarcity of evidence on the RME-only approach, with only one study comparing the RME-plus and RME-only approaches in zones 5 and 6 [30], whereas to the authors’ knowledge, there is no randomized study including zone 4 injuries using the RME-only approach.

The aim of this study was threefold: (1) to gain head-to-head evidence of the RME approach in comparison to EPM with the DEO in terms of mobility, strength, function, and patient satisfaction with the treatment outcomes and the orthosis; (2) to compare outcomes of the RME-only approach with the RME and additional night wrist orthosis approach (RME plus); and (3) to assess the safety of the RME protocol for zone 4 extensor tendon injury repair.

We presumed that the scores of primary and secondary outcomes would be higher in both RME groups compared to the DEO group and similar among the RME-only and RME-plus groups. We expected to find no difference in the complication rate, like possible tendon re-rupture among groups, irrespective of the injured zone or the orthosis used.

## 2. Methods

### 2.1. Study Design

This study was designed as a single-center, prospective, interventional, single-blind, randomized controlled trial conducted from November 2019 to November 2024. It was approved by the Medical Ethics Committee of the Republic of Slovenia on 14 May 2019, approval number 0120-275/2019/6, and conducted according to the Helsinki ethical guidelines [33]. All patients signed the informed consent form before enrolling in the study.

Patients were recruited from the emergency departments of the University Medical Center Maribor (UMC Maribor) and the nearby general hospitals (Ptuj and Slovenj Gradec), while the study was conducted at the rehabilitation unit of the UMC Maribor. The study protocol was presented to the emergency departments of the above-listed hospitals. Once the patients were sent to the rehabilitation unit of the UMC Maribor, they were enrolled in the study if they fulfilled all of the inclusion criteria and excluded if they met one of the exclusion criteria (Table 1). Each patient received detailed oral and written information about the study and the opportunity to discuss the rehabilitation protocol with a physical and rehabilitation medicine (PRM) specialist.

The patients were randomly categorized into one of the three intervention groups receiving the DEO, RME only, or RME plus orthosis. The randomization sequence was created using a web-based program [34] (seed 12165) with a block method (block size 9), with each number representing the type of orthosis the patient obtained, and the consecutive numbers representing the sequential inclusion of the patients in the study (authors’ note: the website used for randomization in 2019 is no longer available). A block size of nine was chosen as the multiplier of the three different orthoses used with the allocation ratio 1:1:1 in a block of nine. The small block size was selected to avoid sample size imbalance in case of interruption. Stratification was not applied. The identical opaque envelopes were sealed and numbered by an investigator (VB), who was not involved in the production of the orthosis or the therapeutic sessions. The assessments were performed by a PRM specialist (VB) or occupational therapist (ZS), who were unaware of the type of orthosis the patient used. The statistician (GV) was not blinded to the meaning of the group labels but was not involved in the data collection procedure.

### 2.2. Interventions

**Figure 1:**
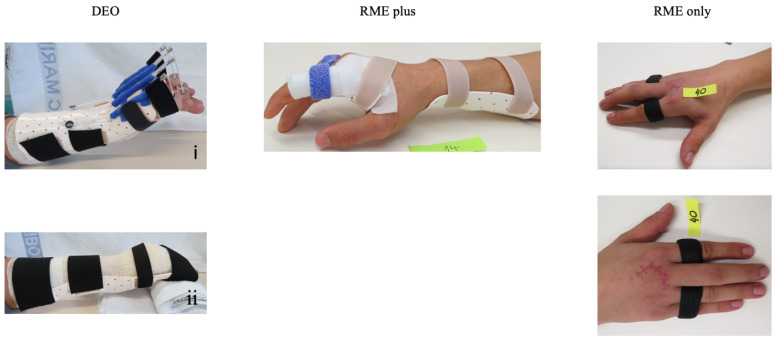
The three types of orthoses used in the study. DEO—the dynamic extension orthosis with (**i**) a dynamic component with a nonelastic circular strip acting as a volar block at the level of the interphalangeal joints and (**ii**) a volar static overnight orthosis; RME plus—the relative motion extension orthosis with the overnight wrist orthosis, RME only—the relative motion extension orthosis.

One of the three types of orthoses was custom-made by the occupational therapists depending on the allocation group (Figure 1).

The DEO consists of a dorsal dynamic component holding the wrist at 30° extension and allowing for passive finger extension at the MCP and IP joints with a recoil of elastic bands. The circular strip attached to the dorsal component acts as a volar block at the level of the IP joints, permitting controlled active finger flexion at the MCP joints to 30–40° within the limits given by the strip. The IP joints are supported in the extended position by the dynamic traction of elastic bands. Patients with the DEO were instructed to perform the exercises within the orthosis. They had to fully flex all their fingers around the MCP joints to the limits set by the volar nonelastic circular strip 3–5 times every half an hour and then let the elastic bands extend the fingers passively. Active extension of the fingers was not allowed. The dorsal dynamic orthosis was removed during the night and replaced by the volar static orthosis. While wearing the DEO, patients could not perform daily activities with the injured hand; they could only use it to fix objects.

The RME-only group received an orthosis that extended the injured finger by 15–20° compared to other MCP joints, while the RME-plus group obtained an additional static overnight wrist orthosis with 10–20° wrist extension. The patients in both groups were instructed to actively flex and extend their fingers as far as the orthosis allowed. They were encouraged to use the injured hand for light daily activities but should not lift or carry loads. Simultaneous wrist and finger flexion was forbidden. No specific instructions on performing regular exercises with the orthosis in place were given since active flexion and extension of the fingers were allowed.

The patients in all three groups were instructed to wear the orthoses continuously for 5 weeks after surgical repair of the tendon. The rehabilitation program was the same for all groups, with once-weekly occupational therapy sessions (OTSs) for 5 weeks while wearing the orthosis and adding once-weekly physiotherapy sessions (PTSs) to occupational therapy sessions after removing the orthosis for another 4 weeks. During the once-weekly OTSs from week 1 to week 5, the correct use of the orthoses, the exercises performed in the DEO, and the activities performed with the RME orthosis were checked. Then, the orthosis was removed to cleanse the skin and orthosis if necessary. The uninjured fingers were passively stretched, and wrist exercises using a tenodesis pattern were performed. After the OTS was completed, the orthosis was placed back on the hand. Five weeks after the injury following orthosis removal, once-weekly OTSs and PTSs were continued. The OTSs aimed to regain hand function with simulated activities of daily living. The PTSs focused on exercises to gradually regain full wrist and finger mobility. Resistance or strength exercises were not performed until 9 weeks after surgical repair.

### 2.3. Outcome Measures

The patients were assessed twice, at 5 (T1) and 9 (T2) weeks after surgical repair of the tendon. The orthosis was removed at T1 by an occupational therapist prior to the first assessment, and the patient was sent to the investigators performing the measurements (occupational therapist (ZS) and/or PRM specialist (VB)) who were blinded to the type of the orthosis the patients used, thereby ensuring a single-blind study.

### 2.4. Primary Outcome Measure

The primary outcome measure was the Jebsen–Taylor hand function test (JTHFT) (Sammons Preston Rolyan Inc., Bolingbrook, IL, USA). It is a timed standardized hand function test developed in 1969 [35]. It assesses functional hand performance with seven subtests simulating unilateral daily activities (writing, simulated page turning, picking up small common objects, simulated feeding, stacking checkers, moving large light objects, and moving large heavy objects). The completion of each activity is measured in seconds. The assessor must be familiar with the test; no special training is required. It has good test–retest and inter-rater reliability, good construct validity (both convergent and divergent), and moderate sensitivity to detect change [36] and determines the MDC_95_ in healthy individuals for the dominant and nondominant hand set within 10.12 and 6.32 s, respectively [37].

### 2.5. Secondary Outcome Measures

Finger range of motion (ROM) was measured using a finger goniometer (Baseline^®^, Fabrication Enterprises, White Plains, NY, USA). The data were given in degrees. Values of total active motion (TAM) for the injured finger were calculated following the Kleinert Verdan protocol using the formula TAM = [(MCP + PIP + DIP flexion) − (MCP + PIP + DIP extension lag)]. The classification was given regarding the contralateral TAM of the uninjured finger where ‘excellent’, ‘good’, ‘adequate’, and ‘poor’ represent equal, more than 75%, more than 50%, and less than 50% ROM of the contralateral finger, respectively [9]. Miller’s criteria were also applied for the classification of finger ROM, where flexion loss is calculated as a difference between composite flexion of the MCP, PIP, and DIP of the contralateral uninjured and injured finger, and extension lag denotes the loss of active composite extension across the MCP, PIP, and DIP of the injured finger. When there is none, <20°, 21–45°, and >45° flexion deficit, Miller’s classification for flexion is excellent, good, fair, and poor, respectively. When assessing active extension lag, none, 5–10°, 11–45°, and >45° represent excellent, good, fair, and poor according to Miller’s classification, respectively [26].

Grip strength of both hands was measured only at T2 with a hand-held hydraulic Jamar^®^ dynamometer (Sammons Preston Rolyan Inc., Bolingbrook, IL, USA) following the Mathiowetz protocol in the standard position (seated in a chair without an armrest with the arm adducted at the shoulder and the elbow at 90° flexion) [38]. The measurements were performed three times for each hand, and the average value was calculated. The value is given as the percentage of the uninjured site.

Two patient-reported outcome measures (PROMs) were used among the participants at T1 and T2. The QuickDASH is a shortened version of the Disability of the Arm, Shoulder, and Hand Questionnaire (DASH) comprising 11 items about upper extremity functionality in the past week, rated by the patient on a Likert scale from 1 to 5. A lower value means a better result. Eight items assess the ability to perform certain upper extremity tasks, and three items assess the patient’s symptoms (pain, tingling, sleep problems). It has good test–retest reliability, good construct validity, high responsiveness [39], a defined minimal detectable change (MDC) of 12.85 points, and a minimal clinically important difference (MCID) of 15.91 points [40]. Official Slovenian translation was used in the study [41]. The translation and cross-cultural adaptation of the DASH (that contains all the items used in the QuickDASH) was performed in a study by Semprimožnik et al. in 2015 [42], and the Slovenian translation and scoring instructions were approved by the Institute for Work and Health, Toronto, Canada.

The Slovenian version of Patient Evaluation Measure (PEM-Slo) is a region-specific PROM for patients with wrist and hand disorders consisting of three parts with a total of 18 items scored on a Likert scale from 1 to 7, with 1 representing the best and 7 the worst score [43]. Values from Part Two and Part Three are summed and expressed as the percentage of the maximum score [44]. It has excellent internal consistency, good test–retest reliability, good construct validity (convergent and divergent), and is highly responsive. Its MDC and MCID are 18.01 and 17.31, respectively [43]. The PEM-Slo is cross-culturally adapted and validated in the Slovenian language [43].

The Modified Orthosis Adherence Questionnaire was administered to patients at T1 to gain information about their habits while wearing the orthosis regarding the removal of the orthosis, time spent without the orthosis, usage of their hand without the orthosis, and for what purpose. The questionnaire is a non-validated, modified questionnaire adapted from the original questionnaire by Sandford et al., who investigated patients’ adherence to orthosis use after tendon injury of the hand [45]. No cross-cultural adaptation into the Slovenian language was performed. However, the results are only descriptive and require no scoring. It was used in other recent studies to evaluate patients’ adherence to wearing different kinds of orthoses [3,30]. The rationale behind its use in our study was to assess adherence, to compare adherence among groups, and to evaluate our results with similar studies.

The visual analog scale (VAS) was used to assess the patients’ comfort while wearing the orthosis separately during the day and night and the ability to use their hands for daily activities with the orthosis in place. The patients marked their comfort on a 100 mm long scale, where 0 meant very uncomfortable or impossible to use the injured hand for daily activities and 100 denoted very comfortable or very easy to use for daily activities. The MCID for patient satisfaction on a 100 mm VAS was determined at 7–11 mm [46].

### 2.6. Statistical Methods with Sample Size Calculation

The sample size was estimated based on the main outcome, i.e., JTHFT total score. Based on a preliminary study and clinical judgment, we assumed that after five and nine weeks, the improvement in the RME-only and RME-plus group would be 6 s better on average compared to the DEO group (e.g., for eight vs. two seconds), and that the standard deviation of the improvement would be 6 in each group. To achieve a statistically significant difference between three equal-size groups using a one-way analysis of variance at the 0.05 alpha level with 80% statistical power, the required sample size was 16 patients in each group. Other randomized controlled studies investigating extensor tendon injuries also included a similar number of patients per group [30,47,48].

The data were collected and analyzed using Microsoft^®^ Excel version 16.48 and IBM^®^ SPSS^®^ Statistics version 29. Descriptive statistics for numerical variables were expressed as means with standard deviations (SDs) and ranges. Categorical variables were presented as frequencies and percentages. Fisher’s exact test and one-way analysis of variance (ANOVA) were applied to evaluate the comparability among the three groups and to assess the adequacy of randomization. Homogeneity of variances for the ANOVA was tested using Levene’s test. Differences in mean values for parameters including the JTHFT, strength, the QuickDASH, PEM, TAM, percentage TAM, Miller’s flexion loss and extension lag, and the VAS were assessed using one-way ANOVA, whereby effect size was estimated using eta-squared (*η*^2^) and the values of 0.01, 0.06, and 0.14 were considered lower limits for small, medium, and large effects, respectively. TAM, Miller’s classification, and Modified Orthosis Adherence Questionnaire scores were analyzed using the extended Fisher’s exact test, whereby effect size was estimated using Cramér’s *V* and the values of 0.1, 0.3, and 0.5 were considered lower limits for small, medium, and large effects, respectively. A *p*-value < 0.05 was considered statistically significant.

## 3. Results

### 3.1. Patient Recruitment

**Figure 2:**
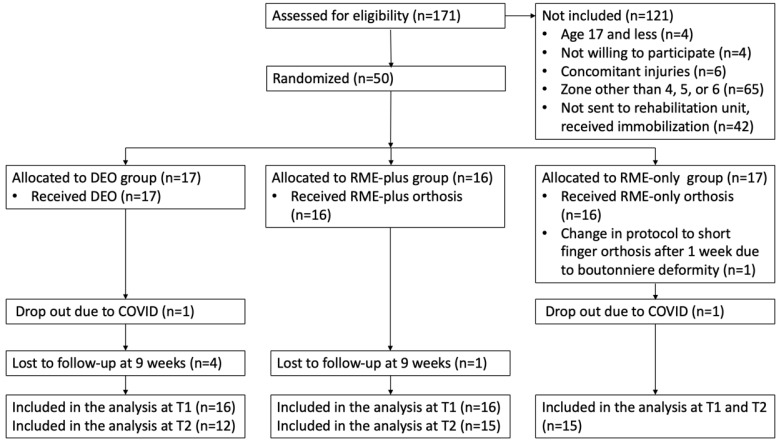
Flowchart of patient recruitment. DEO—dynamic extension orthosis, RME plus—relative motion orthosis and night wrist orthosis, RME only—relative motion orthosis day and night, T1—first assessment, and T2—second assessment.

As seen in the flowchart (Figure 2), there were 171 patients assessed for eligibility, of which 121 were excluded, 4 due to their age being 17 years and below, 4 were not willing to participate, 6 had concomitant injuries, and the majority (65 patients) had injured zones other than 4, 5, or 6. There was also a large group (42 patients) who were not sent to the rehabilitation unit and received immobilization. After fulfilling the inclusion criteria, 50 patients were randomized into one of the three intervention groups, of which 17 were allocated to the DEO group and received the DEO, 16 were allocated to the RME-plus group and received RME plus the orthosis, and 17 were allocated to the RME-only group, of which 1 was excluded from the study due to a change in the rehabilitation protocol for boutonniere deformity. The study was also conducted during the COVID-19 pandemic, and two patients dropped out due to the closure of the rehabilitation unit, one from the DEO group and the other from the RME-only group. Five patients were lost to follow-up after T1 and were assessed only at T1; thereafter, they stopped arriving at the occupational and physical therapy sessions. Four were from the DEO group and one was from the RME-plus group. There were 16 patients from the DEO group included in the analysis at T1 and 12 at T2, 16 from the RME-plus group at T1 and 15 at T2, and 15 from the RME-only group at T1 and T2.

### 3.2. Demographic Characteristics

The patients enrolled in the study were sent to the rehabilitation unit, mainly from the emergency department of the UMC Maribor; only three patients were sent from General Hospital Ptuj and seven from General Hospital Slovenj Gradec. Demographic data and group characteristics are shown in Table 2. Patients were predominantly male (87%), the average age was 43 years, and they were mostly manual workers, with 43% having heavy workloads and an education level of mainly high school or lower. Almost half of the injuries were in zone 5 (47%), and the index finger was the most frequently injured (49%). The injuries occurred predominantly with a sharp object such as glass, work machine, knife (each in 21%), or metal (19%). The non-dominant hand was injured in two-thirds of patients (66%), and most were right-handed (83%). Primary surgical tendon repair was performed on the day of the injury; only three patients had tendon reconstruction the following day and one patient underwent surgery 6 days after the injury. The orthosis was custom-made after surgical repair in 6.4 days on average for all groups; the timespan from injury to orthosis manufacture was not statistically significantly different.

The number of OTSs and PTSs was similar in all groups, albeit slightly higher in the DEO group, but not statistically significantly different. The statistical analysis of the demographic data did not show any statistically significant differences among the three groups regarding age and dominant or non-dominant hand injury, which confirmed that randomization was successful. Patients were, on average, older in the DEO group; however, the difference among groups was not statistically significant.

### 3.3. Primary and Secondary Outcome Measures

Achievements according to the JTHFT, strength, the QuickDASH, and PEM are given in Table 3. The total scores of the JTHFT were better in the RME-only and RME-plus group than in the DEO group; however, the difference was statistically significant at T1 only when the subdomain writing was excluded from the analysis. The times achieved on the JTHFT at T2 were still better in both RME groups compared to the DEO group; however, the differences among groups were not statistically significant. Hand grip strength was measured only at T2, and the results show statistically significantly stronger grip strength in RME groups compared to the DEO group, whereas the results are very similar in both RME groups, as shown in Table 3.

The results of the PROMs show that only QuickDASH scores at T1 were statistically significantly better in both RME groups compared to the DEO group, whereas the values of PEM at T1 and T2, as well as QuickDASH score at T2, do not statistically significantly differ among groups. However, the scores in both RME groups are better than in the DEO group but do not reach statistical significance (Table 3).

The range of motion of the injured fingers given as TAM and the TAM classification is presented in Table 4, and Miller’s criteria for flexion loss and extension lag according to Miller’s classification are shown in Table 5. In all measures of mobility given in degrees, except Miller extension loss, the groups were statistically significantly different, with both RME groups performing better than the DEO group. The differences were generally more significant at T1 than at T2. The patients in the RME-only and RME-plus groups achieved similar results in terms of finger range of motion. Regarding TAM and Miller’s classification, the only statistically significant difference between the groups was in the TAM classification at T1, with the DEO group having the lowest scores, the RME-only group having higher scores, and the RME-plus group having the highest scores. Other classifications (TAM at T2, Miller’s flexion loss, and Miller’s extension lag classification at T1 and T2) were not statistically significantly different among groups.

In the analysis of the Modified Orthosis Adherence Questionnaire (Table 6), only 9% of patients never took off the orthosis. Most patients took it off daily (63%); however, 84% claimed to be without the orthosis for less than 1 h. Most patients drove their car while wearing the orthosis (83%). Seventy percent of patients did not use their hand for daily activities while being without the orthosis. The differences in orthosis adherence were not statistically significant among the three groups.

The patients’ comfort while wearing the orthoses during the day and night did not statistically significantly differ among the groups; however, the highest values denoting higher comfort were reported in the RME-only group for wearing the orthosis during the day (77.9 mm (SD 21.8)) and during the night (81.1 mm (SD 25)), compared to the values of the DEO group of 72.8 mm (SD 27) for day comfort and 68.3 mm (SD 29.6) for night comfort. The most considerable difference was observed when assessing hand usage for daily activities, where values for the RME groups were very close (73 mm (SD 22.1) for the RME-plus group and 72.3 (SD 21.5) for the RME-only group), whereas in the DEO group the VAS score for hand usage was 52.1 mm (SD 34.5) (Table 7).

## 4. Discussion

The outcomes in our study evaluating hand function after extensor tendon injuries in zones 4–6 using three different rehabilitation protocols with the DEO, RME only, and RME plus orthosis indicate that the RME protocol is superior to the DEO protocol as far as range of motion, grip strength, and short-term results according to QuickDASH and JTHFT score are concerned. The RME-plus and RME-only protocols were equivalent, and no tendon ruptures occurred in either of the groups, indicating that the RME-only protocol is safe and non-inferior to the RME-plus protocol.

Higher scores on the JTHFT and QuickDASH point to earlier regain of hand function since the JTHFT simulates the daily activities of the hand, and the QuickDASH gives one’s opinion of hand functionality.

The JTHFT was chosen as the primary outcome measure. Both RME groups achieved better times on the JTHFT compared to the DEO group, especially when the subdomain writing was excluded. The effect size was large (*η*^2^ = 0.14, *p* = 0.034) at T1, but not at T2. Writing is considered a confounding factor, which was also reported in previous studies [37], since it is a typically unilateral dominant hand activity, and the non-dominant hand was injured in 64% of all patients included in the study. The differences among groups at T2 were smaller, indicating a medium effect size (*η*^2^ = 0.12, *p* = 0.090), likely due to the fact that the JTHFT score relies only on the time measured to perform a specific task and may not be sensitive enough to detect subtle differences in hand function. This could be outweighed by using a hand function test that assesses not only the time to complete the test but also the difficulty with which the task is completed and whether the prescribed hand grip is used for the task like the Sollerman hand function test (SHFT) [49], which was used in the study by Collocott et al. [3]. Nevertheless, we decided to use the JTHFT instead of the SHFT since the JTHFT only has seven tasks compared to the SHFT, which assesses twenty activities of daily living, so the assessment could be biased when grading difficulty when performing the test. Another reason for more minor differences among the DEO and RME groups at T2 is that all patients were undertaking OTSs and PTSs after removing the orthosis and started to use their hand for daily activities, whereby they regained hand performance skills. Rehabilitation treatment did not differ among groups; the training of simulated daily activities in OTSs and improvements in mobility of the wrist and fingers in PTSs until week nine were sufficient to lower the differences among the groups at T2. Our observations are similar to those of other studies where short-term differences among groups were more prominent and long-term outcomes were more leveled in other outcome measures when comparing different rehabilitation protocols after extensor tendon injuries [27,47,50,51,52].

When comparing the range of motion according to TAM and Miller’s criteria, the mobility of the injured finger was statistically significantly better at all time points in both RME groups compared to the DEO group, whereas the achievements were similar in the RME groups. The differences among groups were higher at T1, when the %TAM was 78 and 79 for the RME-only and RME-plus groups, respectively, compared to 55 in the DEO group. At T2, both RME groups achieved 93%TAM of the contralateral finger and the DEO group achieved 81%TAM of a contralateral finger. To summarize, at T1, a large effect size was observed for TAM (*η*^2^ = 0.25, *p* < 0.001) and for %TAM of the contralateral digit (*η*^2^ = 0.32, *p* < 0.001). At T2, the differences remained significant but were slightly lower, still showing a large effect size for TAM (*η*^2^ = 0.19, *p* = 0.018). Bühler et al. did not find such differences in their study, with the results for %TAM being similar at 6 and 12 weeks for the DEO and RME-plus groups (79 vs. 82 at 6 weeks and 90 vs. 93 at 12 weeks, respectively) [48].

Grip strength was measured only at T2 because at T1 strenuous activities were forbidden. It was better in both RME groups than in the DEO group, with a large effect size (*η*^2^ = 0.28, *p* = 0.002); these outcomes are better than in the study by Bühler et al. [48]. The RME-only and RME-plus groups in our study reached 5.2 and 8.2 kg (25% and 26%) more, respectively, in terms of grip strength compared to the DEO group, which surpasses the MDC for grip strength set at 5 to 6.5 kg or 19% [53].

The two PROMs used in our study yielded different outcomes, albeit both of them are region-specific outcome measures for the upper extremity; PEM might be even more specific for hand function and handedness addressing the injured hand, whereas the QuickDASH evaluates different daily tasks irrespective of which hand is used for the activity. However, the achievements on PEM did not statistically significantly differ among groups and were all below 18 points, which is the MDC_95_ for PEM. Both RME groups achieved statistically significantly better results on the QuickDASH compared to the DEO group at T1 (large effect size, *η*^2^ = 0.14, *p* = 0.034), whereas QuickDASH scores at T2 and PEM scores at both time points were still higher in both RME groups but not statistically significant (medium effect size, *η*^2^ = 0.12, *p* = 0.086). Differences in QuickDASH scores between the DEO and RME groups at T1 surpassed the MDC_95_ for the QuickDASH, being 16.9 and 14.3 points higher in the RME-only and RME-plus group, respectively, compared to the DEO group. The difference at T2 among groups was lower than 12.85 points, which is the MDC_95_ for the QuickDASH. The QuickDASH scores in a study by Bühler et al. were similar in the DEO and RME-plus groups, which is in line with our results at T2 [48].

Our study’s findings point out earlier regain of hand function in patients using an RME orthosis compared to patients using the DEO. Hall et al. published a similar conclusion in their study comparing three different rehabilitation protocols in which the EAM rehabilitation protocol was considered superior to the immobilization and EPM protocol in terms of regaining better mobility earlier [47]. Bühler et al. concluded in their study that the RME-plus protocol, whereby they used a wrist orthosis in conjunction with the RME orthosis during the day, was non-inferior to the DEO protocol for patients with zone 5 and 6 extensor tendon injuries regarding TAM, QuickDASH score, percentage of grip strength, therapy, and orthotic satisfaction compared to the DEO protocol [48]. The fact that our patients in both RME groups were wearing only an RME orthosis during the day probably explains our findings of the RME protocol being superior to the DEO protocol.

The outcomes of patients in the two RME groups in our study were very similar, as anticipated. Similar results were published by Hirth et al., who compared the “RME day group” wearing an RME orthosis only during the day and a WHFO during the night with the “RME 24 h group” wearing only an RME orthosis continuously. They found no differences between the groups for TAM and Miller’s criteria, grip strength, QuickDASH score, and PRWHE scores [30], which is in line with the results of our study.

Most of the previous research analyzes the outcomes of EAM after extensor tendon injuries in zones 5 and 6, especially with the RME orthosis [3,27,29,30,32], whereas our study also included patients with zone 4 extensor tendon injury. Svens et al. compared RME-plus and RME-only protocols and included zone 4 injuries; nevertheless, only 5% of their sample size was in zone 4 (3 out of 63 patients—2 had RME plus orthosis and 1 had RME only) [54]. Nearly one-third of patients had zone 4 injury in our study, three patients in the DEO group, four in the RME-only group, and six in the RME-plus group. We had to change the treatment protocol for one patient with zone 4 injury in the RME-only group due to the occurrence of boutonniere deformity one week after the injury to the short arc motion protocol with a static orthosis that holds the PIP joint in extension and leaves the MCP and DIP joints free. Besides the one described, no other adverse events occurred when using the DEO and RME orthoses for injuries in zone 4. The sample size of injuries in zone 4 in our study is too small to draw any conclusion about which of the three orthoses used is better; the only conclusions we can make are that both DEO and RME rehabilitation protocols are safe for zone 4 injuries since no ruptures or other adverse events occurred, and that it is essential to gain precise information from the surgeon about the zone that was repaired since skin scars can be a misleading factor when deciding which orthosis to administer.

As seen from the Modified Orthosis Adherence Questionnaire, the differences among groups were not statistically significant (medium effect size, *V* = 0.21, *p* = 0.110). The majority of patients reported removing the orthosis for handwashing and whilst showering. Some of the patients from the DEO group removed the orthosis while dressing, which can be attributed to the cumbersome orthotic design. Most patients were driving their car while wearing the orthoses, mostly the patients in the RME groups, which points to easier use of the hand with the RME orthosis. Reported time spent without the orthosis was usually less than one hour, and only one-third of patients used their hands for daily activities without the orthosis. In the study by Bühler et al., the RME-plus group was more adherent than the group wearing the dynamic wrist–hand–finger orthosis, whereas we found no difference among the groups in our study. However, the most common reasons for orthotic removal in other studies were similar to our results, such as handwashing, showering, and dressing [30,48].

When assessing patient comfort while wearing the orthosis using a VAS, we anticipated the highest values in the RME groups since the RME orthosis is small and therefore easier to wear, and the patients were allowed to use their hand for light daily activities, whereas the DEO is more bulky and it restrains hand usage in daily activities. The absolute values were higher in the RME groups, especially when assessing hand usage and comfort at night, where the values surpassed the MDC for the VAS measuring patient satisfaction set at 7–11 mm [46]. Nevertheless, the differences among groups were not statistically significant (small effect size, *η*^2^ = 0.01, *p* = 0.837 for daytime comfort; medium effect size, *η*^2^ = 0.06, *p* = 0.342 for nighttime comfort; medium effect size, *η*^2^ = 0.12, *p* = 0.081 for hand usage with orthosis), whereas in the study by Collocott, patients with the RME orthosis reported higher satisfaction with the orthosis compared to the group wearing a static orthosis that enabled EAM [3].

No tendon ruptures were observed in any groups, meaning that all of the orthoses used in this study represent a safe postoperative rehabilitation protocol. We have included, however, only isolated lacerations of one tendon for easier comparison among the groups, whereas the RME orthoses can also be used for up to three lacerations [26], mostly two injured extensor tendons in zones 5 and 6 [3,27,30,54].

The results of our study indicate that the preferable choice of orthosis after extensor tendon injuries in zones 4–6 is an RME orthosis before a DEO when aiming to regain hand function early and that the RME-only protocol is as safe and effective as the RME-plus protocol for zone 4 injuries.

### Limitations of the Study

One of this study’s shortcomings is the lower number of subjects included. The sample size was estimated on relatively realistic assumptions about the primary outcome. Naturally, this study may have, therefore, been underpowered regarding some secondary outcomes, but the results indicate that many statistically significant differences were nevertheless observed. Still, the number of subjects in each group was just enough to meet the calculated sample size, whereas the drop-out rate was not considered in the calculation. We actively encouraged the patients to finish the study protocol by phone if they missed an OTS or PTS appointment. However, some subjects did not respond to our invitations over the phone, and the drop-out rate should have been incorporated into the sample size calculation. We believe the relatively small sample size does not compromise this study’s validity. Nevertheless, the generalizability of a more extensive study (or a future meta-analysis in which our trial could be included) would, of course, be more outstanding.

Another obstacle in patient recruitment and leading to the consequential smaller sample size was the non-referral of patients from the emergency units to the rehabilitation unit in the first days following injury of the extensor tendon, which is in line with the findings of Hirth et al. who stated that surgeon and clinic preferences about postoperative splinting protocol are the primary barriers in implementing the RME approach [32]. Many patients who met the inclusion criteria surpassed the study enrolment process, coming to our unit as late as 4 weeks following injury and wearing static splints continuously for 4 weeks. As seen in the flowchart of patient recruitment in Figure 2, 42 patients eligible to participate in the study were still managed with immobilization after extensor tendon repair in zones 4–6. Despite abundant scientific evidence about the harm of immobilization for tendon healing and adhesion formation [6,17,18,19,20], the standard postoperative protocol for extensor tendon injury at our center in 2018 was still immobilization [55]. Despite the above-stated shortcomings in the recruitment process, we included a comparable number of subjects as in some other randomized studies about extensor tendon injuries [30,47,48].

Another shortcoming of this study was the longer study duration than initially planned. It was abruptly interrupted in March 2020 when the local government closed down the rehabilitation unit due to the COVID-19 pandemic, and accessibility to the outpatient rehabilitation unit was significantly hindered [56]. Fortunately, only two patients dropped out during the pandemic; nevertheless, no new patients were recruited for about a year. We believe that the interruption caused by the pandemic did not impact the study’s validity since the rehabilitation protocols were not altered once the study was resumed. The two dropped-out patients were not included in the analysis.

Adherence to wearing the orthosis was assessed with the Modified Orthosis Adherence Questionnaire. All patients in the DEO group reported removing the orthosis daily, which could have also been performed to replace the DEO for night volar static orthosis. Since the investigator was not supervising or checking the answers given by patients, they could have reported the necessary evening removal as non-adherence. Overall, no tendon ruptures occurred in the study, which indirectly indicates that patients were generally adherent to treatment.

Our study did not observe the return-to-work timeline since it can often be influenced by the patient’s motivation to return to work rather than the actual sequelae of the extensor tendon injury. Our insurance system reimburses 100% of the salary when on sick leave if the injury occurred at the workplace. Many workers may be less motivated to return to work early due to the stated benefits.

## 5. Conclusions

The EAM rehabilitation protocol after extensor tendon injuries in zones 4–6 with the RME concept is superior to the EPM protocol with the DEO regarding the earlier regain of hand function in terms of mobility, strength, JTHFT score, and QuickDASH score. Short-term differences after 5 weeks were higher than long-term differences after 9 weeks. The DEO and RME protocols were equivalent regarding patients’ adherence and satisfaction with the orthosis. We found no differences in the RME-plus and RME-only protocols, indicating the safe use of the RME-only protocol in single extensor tendon injuries in zones 4–6.

## Figures and Tables

**Table 1:** Inclusion and exclusion criteria.

Inclusion Criteria	Exclusion Criteria
More than 50% of transverse or oblique single extensor tendon lacerations in zones 4, 5, or 6	Injury to extensor tendons in zones 1, 2, 3, 7, or 8 and injury to thumb extensor tendon
Surgical reconstruction of the tendon	Injury to more than one extensor tendons in zones 4, 5, or 6
10 or less days since surgical reconstruction of the tendon	Concomitant injuries (bone fractures, extensive laceration of large area, associated flexor tendon injuries, etc.)
No hand health conditions	Associated diseases of the hands (rheumatoid arthritis, osteoarthritis with small joint deformities, Dupuytren’s contracture, small joint contractures after an old injury, a neurologic disease with associated weakness, paresis, or spasticity of the hand, etc.)
No contralateral finger amputation	Amputation of the contralateral finger at any level
Patient’s willingness to participate in the study and ability to give informed consent	Patient not willing to participate in the study or not being able to give informed consent (e.g., cognitive impairment)
Age 18 years and above	Age 17 years or less

**Table 2:** Demographics and patient characteristics.

Variable	DEO Group	RME-Only Group	RME-Plus Group	Combined Data for All Groups
Number of patients	16	15	16	47
- Male, n (%)	13 (81)	12 (80)	16 (100)	41 (87)
Age * in years, mean (SD) [range]	48.9 (14.8) [21–71]	42.5(10.7) [21–61]	39.3 (15.3) [19–67]	43.6 (14.1) [19–71]
Education level, n (%)				
- high school or lower	13 (81)	10 (67)	11 (69)	34 (72)
- university or higher	1 (6)	4 (27)	5 (31)	10 (21)
- unknown	2 (13)	1(6)	0 (0)	3 (6)
Type of work, n (%)				
- light	5 (31)	6 (40)	4 (25)	15 (32)
- medium	5 (31)	4 (27)	3 (19)	12 (26)
- heavy	6 (38)	5 (33)	9 (56)	20 (43)
Dominance, n (%)				
- right	12 (75)	14 (93)	13 (81)	39 (83)
- left	4 (25)	1 (7)	3 (19)	8 (17)
Dominant hand injured **, n (%)	6 (38)	5 (33)	5 (31)	16 (34)
Mechanism of injury, n (%)				
- sharp	15 (94)	15 (100)	14 (88)	44 (94)
- crush	1 (8)	0 (0)	1 (6)	2 (4)
- blunt	0 (0)	0 (0)	1 (6)	1 (2)
Cause of injury, n (%)				
- glass	1 (6)	5 (33)	4 (25)	10 (21)
- work machine	4 (25)	3 (20)	3 (19)	10 (21)
- knife	4 (25)	3 (20)	3 (19)	10 (21)
- metal	3 (19)	2 (13)	4 (25)	9 (19)
- saw	0 (0)	2 (13)	0 (0)	2 (4)
- other	4 (25)	0 (0)	2 (13)	6 (13)
Finger injured, n (%)				
- index	5 (31)	10 (67)	8 (50)	23 (49)
- middle	7 (44)	1 (7)	4 (25)	12 (26)
- ring	2 (13)	0 (0)	2 (13)	4 (9)
- small	2 (13)	4 (27)	2 (13)	8 (17)
Zone injured, n (%)				
- 4	5 (31)	5 (33)	6 (38)	16 (34)
- 5	8 (50)	7 (47)	7 (44)	22 (47)
- 6	3 (19)	3 (20)	3 (19)	9 (19)
Days from injury to tendon repair ***, mean (SD) [range]	0 (0.2) [0–1]	0 (0) [0]	0.5 (1.5) [0–6]	0.2 (0.9) [0–6]
Days from tendon repair to therapy with orthosis ****, mean (SD) [range]	7.2 (1.5) [4–10]	6.5 (2.4) [2–10]	5.6 (2.8) [1–10]	6.4 (2.3) [1–10]
Number of OTSs *†, mean (SD) [range]	8.9 (4.5) [4–24]	7.4 (0.8) [6–8]	7.5 (1.1) [4–8]	7.9 (2.7) [4–24]
Number of PTSs *††, mean (SD) [range]	6.0 (5.7) [0–20]	3.8 (2.5) [0–12]	4.1 (2.4) [0–9]	4.6 (3.9) [0–20]

DEO—dynamic extension orthosis, RME plus—relative motion orthosis and night wrist orthosis, RME only—relative motion orthosis day and night, n—number, SD—standard deviation, OTS—occupational therapy session, and PTS—physical therapy session. * *p* = 0.146 (one-way ANOVA), ** *p* = 0.730 (Fisher’s exact test), *** *p* = 0.240 (one-way ANOVA), **** *p* = 0.138 (one-way ANOVA),*† *p* = 0.268 (one-way ANOVA), and *†† *p* = 0.255 (one-way ANOVA).

**Table 3:** Values achieved on JTHFT, hand grip strength measurements, QuickDASH, and PEM.

Outcome Measure	DEO Group	RME-Only Group	RME-Plus Group	Combined Results for All Groups	*p*	Effect Size (*η*^2^) ***
JTHFT total in s, mean (SD) [range]						
- T1	70.5 (20.9) [43.4–127.7]	62.8 (19.2) [38.6–109]	62.5 (15.5) [39.5–94.5]	64.5 (18.8) [38.6–127.7]	0.397 *	0.04
- T1 w/o writing	42.1 (13.2) [30–83.9]	33.6 (6.3) [26–48]	34.9 (7.3) [24.6–51.6]	36.6 (13.1) [24.6–83.9]	0.034 *	0.14
- T2	59.3 (16.6) [36.1–95.1]	54.1 (13.9) [32.9–73.4]	54.7 (14.3) [35.3–76.4]	55.8 (14.6) [32.9–95.1]	0.632 *	0.02
- T2 w/o writing	34.8 (8.4) [24.5–57.6]	29.9 (3.9) [22.6–36]	30.2 (5.6) [23.7–44.3]	31.4 (6.3) [22.6–57.6]	0.090 *	0.12
Strength in kg, mean (SD) [range]						
- Injured hand	27.1 (12.7) [8.6–46]	32.3 (15.9) [17.6–53]	35.3 (9.5) [19.3–52.6]	31.8 (18.9) [8.6–53]		
- Healthy hand	45.5 (6.4) [34.3–53.6]	39.7 (11.8) [19–64]	42.3 (8.4) [29.6–63.3]	42.3 (9.4) [19–64]		
- Ratio injured/healthy hand	0.58 (1.22) [0.19–0.89]	0.83 (1.16) [0.57–1.06]	0.84 (3.27) [0.49–1.22]	0.76 (4.22) [0.19–1.22]	0.002 **	0.28
QuickDASH score at T1 (SD) [range]	50.7 (18.7) [9.1–81.8]	33.8 (19.8) [0–65.9]	36.4 (18.3) [4.5–61.4]	40.4 (20) [0–81.8]	0.034 **	0.14
QuickDASH score at T2 (SD) [range]	27.2 (23.5) [0–77.3]	15.8 (15.3) [0–43.2]	12.8 (11.7) [0–38.6]	18 (17.6) [0–77.3]	0.086 **	0.12
PEM score at T1 (SD) [range]	36.9 (19.7) [2.6–71.8]	32.2 (20.1) [0–66.7]	28.6 (13.5) [2.6–48.7]	32.6 (17.9) [0–71.8]	0.429 **	0.04
PEM score at T2 (SD) [range]	26.4 (21.4) [1.3–66.7]	14.4 (13.3) [0–42.3]	15.3 (11.6) [0–38.5]	18.1 (16) [0–66.7]	0.106 **	0.11

DEO—dynamic extension orthosis, RME plus—relative motion orthosis and night wrist orthosis, RME only—relative motion orthosis day and night, SD—standard deviation, T1—first assessment at 5 weeks, T2—second assessment at 9 weeks, w/o—without, QuickDASH—shortened version of Disability of the Arm, Shoulder, and Hand Questionnaire, and PEM—Patient Evaluation Measure. * one-way ANOVA (assumption of homogeneity of variances was met in all cases: *p*-values for Levene’s test were ≥0.4). ** one-way ANOVA (assumption of homogeneity of variances was met in all cases: *p*-values for Levene’s test were ≥0.1). *** see Section 2.6 for interpretation guidelines.

**Table 4:** Range of motion of injured and contralateral finger according to TAM classification.

Range of Motion	DEO Group	RME-Only Group	RME-Plus Group	Combined Results for All Groups	*p* * p **	Effect Size ***
T1 TAM ° injured digit, mean (SD) [range]	146.6 (58.6) [20–260]	205.6 (60.2) [150–395]	207.5 (31.4) [160–265]	186.2 (58.1) [20–395]	* 0.002	*η*^2^ = 0.25
T1 TAM ° contralateral digit, mean (SD) [range]	264.4 (12.4) [245–285]	262.0 (34.1) [210–370]	263.4 (18.9) [235–295]	263.3 (22.8) [210–370]		
T1% TAM of contralateral digit	55 (22) [8–96]	78 (16) [51–107]	79 (11) [65–102]	71 (20) [8–107]	* <0.001	*η*^2^ = 0.32
T1 TAM classification, n (%)					** 0.009	*V* = 0.25
- excellent	1 (6)	2 (13)	2 (13)	5 (11)		
- good	2 (13)	5 (33)	9 (56)	16 (34)		
- adequate	7 (44)	8 (53)	5 (31)	20 (43)		
- poor	6 (38)	0 (0)	0 (0)	6 (13)		
T2 TAM ° injured digit, mean (SD) [range]	217.1 (42.7) [130–265]	243.7 (25) [205–300]	249.7 (19.6) [205–280]	238.2 (32) [130–300]	* 0.018	*η*^2^ = 0.19
T2 TAM ° contralateral digit, mean (SD) [range]	265.4 (19.1) [225–295]	262.3 (18.9) [215–285]	268.7 (17.3) [235–300]	265.5 (18.1) [215–300]		
T2% TAM of contralateral digit	81 (16) [47–102]	93 (11) [72–113]	93 (8) [77–104]	90 (12) [47–113]	* 0.022	*η*^2^ = 0.18
T2 TAM classification, n (%)					** 0.198	*V* = 0.18
- excellent	3 (25)	8 (53)	10 (67)	21 (50)		
- good	6 (50)	6 (40)	5 (33)	17 (41)		
- adequate	2 (17)	1 (7)	0 (0)	3 (7)		
- poor	1 (8)	0 (0)	0 (0)	1 (2)		

* one-way ANOVA (assumption of homogeneity of variances was met in all cases: *p*-values for Levene’s test were ≥0.1). ** extended Fisher’s exact test. DEO—dynamic extension orthosis, RME plus—relative motion orthosis and night wrist orthosis, RME only—relative motion orthosis day and night, TAM—total active motion, SD—standard deviation, T1—first assessment at 5 weeks, and T2—second assessment at 9 weeks. *** see Section 2.6 for interpretation guidelines.

**Table 5:** Range of motion of injured and contralateral finger according to Miller’s criteria for flexion loss and extension lag.

Range of Motion	DEO Group	RME-Only Group	RME-Plus Group	Combined Results for All Groups	*p* * p **	Effect Size ***
Miller’s criteria in °, mean (SD) [range] T1 flexion loss	106.3 (46.8) [10–175]	54.3 (31.6) [0–100]	42.8 (25.2) [0–75]	68.1 (44.9) [0–175]	* <0.001	*η*^2^ = 0.39
T1 Miller’s flexion loss classification, n (%)					** 0.131	*V* = 0.18
- excellent	0 (0)	1 (7)	2 (13)	3 (6)		
- good	1 (6)	2 (13)	2 (13)	5 (11)		
- fair	1 (6)	5 (33)	5 (31)	11 (23)		
- poor	14 (88)	7 (47)	7 (44)	28 (60)		
Miller’s criteria in °, mean (SD) [range] T1 extension lag	12.8 (18) [0–55]	6.0 (9.7) [0–25]	13.8 (13.7) [0–40]	11(14.4) [0–55]	* 0.272	*η*^2^ = 0.06
T1 Miller’s extension lag classification, n (%)					** 0.187	*V* = 0.18
- excellent	9 (56)	10 (67)	5 (31)	24 (51)		
- good	1 (6)	2 (13)	3 (19)	6 (13)		
- fair	4 (25)	3 (20)	8 (50)	15 (32)		
- poor	2 (13)	0 (0)	0 (0)	2 (4)		
Miller’s criteria in °, mean (SD) [range] T2 flexion loss	40.8 (33.3) [0–100]	19.0 (21.6) [0–70]	16.0 (17.2) [0–50]	24.2 (24.9) [0–100]	* 0.018	*η*^2^ = 0.19
T2 Miller’s flexion loss classification, n (%)					** 0.459	*V* = 0.16
- excellent	1 (8)	5 (33)	4 (27)	8 (19)		
- good	3 (25)	4 (27)	7 (47)	10 (24)		
- fair	4 (33)	4 (27)	2 (13)	14 (33)		
- poor	4 (33)	2 (13)	2 (13)	10 (24)		
Miller’s criteria in °, mean (SD) [range] T2 extension lag	6.6 (14.7) [0–45]	2.0 (4.1) [0–10]	5.0 (7.6) [0–25]	4.4 (9.1) [0–45]	* 0.402	*η*^2^ = 0.05
T2 Miller’s extension lag classification, n (%)					** 0.560	*V* = 0.14
- excellent	8 (67)	12 (80)	9 (60)	29 (69)		
- good	2 (17)	3 (20)	4 (27)	9 (21)		
- fair	2 (17)	0 (0)	2 (13)	4 (10)		
- poor	0 (0)	0 (0)	0 (0)	0 (0)		

* one-way ANOVA (assumption of homogeneity of variances was met in all cases: *p*-values for Levene’s test were ≥0.1). ** extended Fisher’s exact test. DEO—dynamic extension orthosis, RME plus—relative motion orthosis and night wrist orthosis, RME only—relative motion orthosis day and night, SD—standard deviation, T1—first assessment at 5 weeks, and T2—second assessment at 9 weeks. *** see Section 2.6 for interpretation guidelines.

**Table 6:** Modified Orthosis Adherence Questionnaire.

Adherence Variable	DEO Group	RME-Only Group	RME-Plus Group	Combined Data for All Groups	*p* *	Effect Size *(V)* **
Removal of orthosis, n (%)					0.110	0.21
- yes	16 (100)	12 (80)	15 (94)	43 (92)		
- no	0 (0)	3 (20)	1 (6)	4 (9)		
Frequency of orthosis removal, n (%)					0.045	0.20
- never	0 (0)	2 (14)	0 (0)	2 (4)		
- only once	1(6)	1 (7)	1 (6)	3 (7)		
- 2–6 times	1 (6)	4 (29)	7 (44)	12 (26)		
- every day	14 (88)	7 (50)	8 (50)	29 (63)		
Time spent w/o orthosis, n (%)					0.769	0.09
- Less than 1 h	13 (81)	11 (92)	13 (81)	37 (84)		
- More than 1 h	3 (19)	1 (8)	3 (19)	7 (16)		
Using hand for DA w/o orthosis, n (%)					1.000	0.04
- yes	4 (27)	5 (33)	5 (31)	14 (30)		
- no	11 (73)	10 (67)	11 (69)	32 (70)		
Car driving with orthosis, n (%)					0.138	0.21
- yes	10 (67)	13 (87)	15 (94)	38 (83)		
- no	5 (33)	2 (13)	1 (6)	8 (17)		

* extended Fisher’s exact test. ** see Section 2.6 for interpretation guidelines. DEO—dynamic extension orthosis, RME plus—relative motion orthosis and night wrist orthosis, RME only—relative motion orthosis day and night, DA—daily activity, n—number, and w/o—without.

**Table 7:** Patients’ comfort while wearing the orthosis and their ability to use their hands with the orthosis in place, assessed on a 100 mm visual analog scale (VAS).

VAS	DEO Group	RME-Only Group	RME-Plus Group	Combined Data for All Groups	*p* *	Effect Size (*η*^2^) **
VAS comfort during day in mm, mean (SD) [range]	72.8 (27) [13–100]	77.9 (21.8) [42–100]	76.3 (16.1) [50–100]	75.7 (21.4) [13–100]	0.837	0.01
VAS comfort at night in mm, mean (SD) [range]	68.3 (29.6) [23–100]	81.1 (25.0) [49–100]	79.0 (19.4) [22–100]	76.5 (23.1) [22–100]	0.342	0.06
VAS hand usage in mm, mean (SD) [range]	52.1 (34.5) [0–100]	72.3 (21.5) [28–100]	73 (22.1) [31–100]	66.2 (27.6) [0–100]	0.081	0.12

* one-way ANOVA. ** see Section 2.6 for interpretation guidelines. VAS—100 mm visual analog scale, DEO—dynamic extension orthosis, RME plus—relative motion orthosis and night wrist orthosis, RME only—relative motion orthosis day and night, SD—standard deviation, and mm—millimeter.

## Data Availability

Dataset available on request from the authors.

## Abbreviations

EAM	early active motion
EPM	early passive motion
DEO	dynamic extension orthosis
RME	relative motion extension
MCP	metacarpophalangeal
IP	interphalangeal
PIP	proximal interphalangeal
DIP	distal interphalangeal
EDC	extensor digitorum communis muscle
WHFO	wrist–hand–finger orthosis
JTHFT	Jebsen–Taylor hand function test
TAM	total active motion
PROM	Patient Reported Outcome Measure
QuickDASH	shortened version of Disability of the Arm, Shoulder, and Hand Questionnaire
PEM	Patient Evaluation Measure
PRWHE	Patient Wrist and Hand Evaluation
MDC	minimal detectable change
MCID	minimal clinically important difference
ROM	range of motion
VAS	visual analog scale
OTS	occupational therapy session
PTS	physical therapy session
